# Trends in stroke incidence among elderly low-income residents of rural China: a population-based study from 1992 to 2016

**DOI:** 10.18632/aging.101657

**Published:** 2018-11-28

**Authors:** Hongyan Lu, Zaiyu Guo, Jie Liu, Heliang Zhang, Wei Zhao, Yanan Wu, Jingxian Ni, Wei Liu, Jun Tu, Jinghua Wang, Xianjia Ning, Jianning Zhang

**Affiliations:** 1 Department of Neurology, Tianjin Medical University General Hospital, Tianjin 300052, China; 2 Tianjin Neurological Institute, Key Laboratory of Post-Neuroinjury Neuro-repair and Regeneration in Central Nervous System, Ministry of Education and Tianjin City, Tianjin 300052, China; 3 Department of Neurology, Tianjin TEDA Hospital, Tianjin 300457, China; 4 Laboratory of Epidemiology, Tianjin Neurological Institute, Tianjin 30005, China; 5 Department of Neurosurgery, Tianjin Medical University General Hospital, Tianjin 300052, China; * Equal contribution

**Keywords:** stroke, incidence, epidemiology, elderly, trends

## Abstract

In China, >70% of stroke deaths occur in people aged ≥65 years. However, trends in the stroke incidence among elderly people are unclear. We aimed to determine trends in the stroke incidence among elderly people in rural China. This was a population-based surveillance study conducted in Tianjin, China. Stroke events and all deaths were registered annually. Trends and annual proportion of change in incidence of first-ever stroke were evaluated from 1992 to 2016. The age-standardized incidence of first-ever stroke increased annually by 3.7% overall in elderly people (2.7% for men; 5.0% for women; all P<0.05). However, from 2008 to 2016, there was no significant change in the trends of stroke incidence among elderly people, across gender and subtypes. The proportion of elderly patients with first-ever stroke decreased by 1.1% annually. In contrast to young patients, annual changes in the incidence of stroke tended to be slight in elderly patients (3.7% vs. 9.5%) with greater increase in female patients than those in male patients (2.7% vs. 10.3% for men; 5.0% vs. 8.9% for women). Thus, the control of risk factors for stroke among elderly people is crucial, especially among older women, to reduce the burden of stroke in China.

## Introduction

Stroke was the leading disease burden worldwide in 2013 [1]. The age-standardized incidence of stroke in high-income countries decreased by 12% between 1990 and 2010, while that in low- and middle-income countries increased by 12% [2].

The current prevalence of stroke in China appears to be the highest among low- and middle-income countries (536–1040/100,000) [3–6]. The National Epidemio-logical Study of Stroke in China showed that the age-standardized prevalence, incidence, and mortality rates of stroke in China in 2012–2013 were 1115/100,000; 247/100,000; and 115/100,000; respectively [7]. The study on the global burden of disease conducted in 2013 showed that >70% of the stroke deaths occurred in people aged ≥65 years in China [8].

The incidence of stroke among rural residents was significantly higher than that among urban residents in China [7]. However, there have been few recent studies on the trends of stroke incidence in the elderly population, especially in rural low-income populations.

In the present study, we aimed to explore the trends in the incidence of first-ever stroke among elderly people aged ≥65 years in rural China from 1992 to 2016.

## RESULTS

### Characteristics of elderly patients with first-ever stroke

During the 362,596 person-years of follow-up in this study, 1188 patients experienced stroke overall, including 709 (59.7%) male patients. Patients aged ≥65 years, with an average age of 65.35 years (65.24 years in men and 65.52 years in women), accounted for 52.3% (621 cases). The average duration of education for stroke patients was 3.36±3.28 years overall (5.17±3.07 and 1.71±2.50 years for those aged <65 and ≥65 years, respectively). The education duration (equivalent to elementary school level) was low both in patients aged <65 years and ≥65 years (5.2 years vs. 1.7 years), although it was significantly higher in patients aged <65 years than that in patients aged ≥65 years. The proportion of diagnosis by imaging (computerized tomography [CT]/magnetic resonance imaging [MRI]) improved from 1992 to 2016 (65.3% to 88.5%), but the rate of diagnosis via imaging was significantly lower in patients aged ≥65 years (67.3%) than in patients aged <65 years (92.9%). Young patients were more likely to have a higher prevalence of diabetes (14.3% vs 9.8%; P=0.018) and current alcohol consumption (31.5% vs 17.0%; P<0.001) than elderly patients. Moreover, the current smoking rate in men was higher in young patients than in elderly patients (72.6% vs 58.9%; P<0.001), but a reverse trend was found in women (1.8% vs 6.2%, P=0.014; Table 1).

**Table1:** The descriptive characteristics of patients with first-ever stroke in this study by sex and periods.

**Characteristics**	**Men**				**Women**				**Total**		
	**≥65 years**	**<65 years**	**Overall**		**≥65 years**	**<65 years**	**Overall**		**≥65 years**	**<65 years**	**Overall**
**Cases, n (%)**	371 (52.3)	338 (47.7)	709 (59.7)		250 (52.2)	229 (47.8)	479 (40.3)		621 (52.3)	567 (47.7)	1188 (100)
**Person-year**	19393	169189	188582		19889	154085	173974		39282	323314	362596
**Age of onset, mean (SD), year**											
	74.03 (6.13)	55.60 (6.90)	65.24 (11.28)		75.02 (6.28)	55.14 (7.50)	65.52 (12.09)		74.43 (6.21)	55.41 (7.15)	65.35 (11.61)
**Education level, mean (SD), year:**											
	2.12 (2.67)	5.86 (2.88)	3.90 (3.34)		1.09 (2.07)	4.15 (3.07)	2.56 (3.01)		1.71 (2.50)	5.17 (3.07)	3.36 (3.28)
**Diagnosis by CT/MRI, n (%)**											
	247 (66.6)	313 (92.6)	560 (79.0)		171 (68.4)	214 (93.4)	385 (80.4)		418 (67.3)	527 (92.9)	945 (79.5)
**Hypertension, n (%): n=1176**											
	527 (88.6)	508 (89.9)	1035 (88.0)		310 (84.7)	301 (89.3)	611 (86.9)		527 (88.6)	508 (89.9)	1035 (88.0)
**DM, n (%): n=1163**											
	24 (6.6)	35 (10.4)	59 (8.4)		35 (14.6)	45 (20.1)	80 (17.2)		59 (9.8)	80 (14.3)	139 (12.0)
**Hyperlipermia, n (%): n=1138**											
	35 (9.8)	31 (9.5)	69 (9.7)		27 (11.6)	43 (19.3)	74 (15.4)		62 (10.5)	74 (13.5)	143 (12.0)
**Current smoking, n (%): n=1171**											
	215 (58.9)	244 (72.6)	459 (65.5)		15 (6.2)	4 (1.8)	19 (4.0)		230 (37.8)	248 (44.0)	478 (40.8)
**Current alcohol consumption, n (%): n=1171**											
	102 (27.9)	176 (52.5)	278 (39.7)		2 (0.8)	1 (0.4)	3 (0.6)		104 (17.1)	177 (31.5)	281 (24.0)

### Trends in the incidence of first-ever stroke among elderly patients by sex and stroke subtype

The age-standardized incidence of first-ever stroke per 100,000 person-years was 970.44 in 1992 and 2349.35 in 2016 in those aged ≥65 years. Regarding the stroke subtype, the corresponding incidence rates in 1992 and 2016 were 426.13 and 285.11 for intracerebral hemorrhage (ICH) and 544.31 and 1888.90 for ischemic stroke (IS), respectively.

From 1992 to 2016, the age-standardized incidence of stroke in elderly patients increased by 3.7% (95% confidence interval [CI]: 1.4, 6.0) per year overall: 2.7% (95% CI: 0.9, 4.6) for men; and 5.0% (95% CI: 2.1, 7.9) for women (all P<0.05). More specifically, the age-standardized incidence significantly increased for IS both in men and women aged ≥65 years (total, 5.5%; men, 4.5%; and women, 6.8%; all P<0.05). For ICH, there were no significant trends in age-standardized incidence (all P>0.05).

However, from 2008 to 2016, there was no significant change in the trends of stroke incidence among elderly patients, across gender and subtypes (Table 2).

**Table 2:** Trends in incidence of first-ever stroke per 100000 person-year in elderly population during 1992 to 2016 by sex and subtypes.

**Year**	**ICH**				**IS**				**Total**		
	**Men**	**Women**	**Total**		**Men**	**Women**	**Total**		**Men**	**Women**	**Total**
**1992**	583.15(87.34)	257.99(59.69)	426.13(53.50)		583.15(87.34)	501.66(83.22)	544.31(60.46)		1166.30(123.48)	759.65(102.39)	970.44(80.72)
**1993**	719.13(97.82)	234.90(56.18)	478.74(56.58)		810.07(103.82)	212.34(53.42)	512.61(58.54)		1529.20(142.59)	447.25(77.52)	991.15(81.38)
**1994**	236.63(56.39)	0	116.69(27.91)		851.70(106.95)	849.71(106.11)	850.17(75.30)		1088.33(120.88)	849.71(106.11)	966.85(80.30)
**1995**	344.09(67.90)	129.79(40.74)	227.40(38.57)		615.33(90.79)	533.21(82.55)	583.00(61.75)		959.43(113.35)	663.00(92.05)	810.41(72.80)
**1996**	616.29(90.74)	130.33(40.54)	370.17(49.00)		808.62(103.93)	522.85(81.18)	658.30(65.34)		1424.91(137.92)	653.19(90.73)	1028.48(81.65)
**1997**	360.83(69.35)	0	176.32(33.74)		698.66(96.48)	0	335.25(46.51)		1059.48(118.79)	0	511.57(57.45)
**1998**	463.89(78.52)	130.87(40.37)	287.21(42.97)		976.70(113.91)	84.15(32.37)	527.96(58.25)		1440.59(138.31)	215.03(51.74)	1003.61(80.30)
**1999**	740.07(98.84)	130.33(40.18)	431.73(52.53)		818.56(103.94)	130.33(40.18)	460.59(54.25)		1558.64(143.38)	260.66(56.83)	893.32(75.54)
**2000**	125.39(40.30)	261.21(56.71)	191.91(34.80)		1554.78(141.80)	996.33(110.72)	1268.12(89.42)		1680.17(147.40)	1257.54(124.37)	2792.66(132.59)
**2001**	224.34(53.76)	211.46(50.87)	216.94(36.90)		1173.57(122.90)	602.46(85.85)	880.78(74.32)		1397.91(134.12)	813.93(99.77)	1097.72(82.97)
**2002**	97.77(35.36)	473.04(76.03)	279.77(41.81)		765.01(98.87)	503.00(78.40)	628.07(62.63)		862.78(104.99)	976.04(109.18)	907.84(75.29)
**2003**	124.89(39.83)	421.63(71.69)	278.84(41.65)		1727.43(148.03)	923.21(106.06)	1330.27(90.92)		1852.32(153.27)	1344.84(127.98)	1609.10(99.98)
**2004**	591.30(86.33)	340.40(64.15)	468.27(53.75)		1094.27(117.41)	996.14(109.70)	1013.14(79.04)		1685.57(145.67)	1336.54(127.04)	1490.41(95.85)
**2005**	125.39(39.94)	133.38(40.38)	323.15(44.88)		1515.29(138.74)	823.54(100.30)	1140.99(84.29)		1640.67(144.36)	956.91(108.11)	1270.25(88.93)
**2006**	95.03(34.81)	0	42.80(16.36)		632.43(89.79)	290.57(59.70)	452.32(53.17)		727.46(96.29)	290.57(59.70)	495.12(55.63)
**2007**	733.69(96.89)	480.35(76.61)	582.86(60.36)		1681.81(146.62)	1504.29(135.51)	1597.59(99.88)		2415.52(175.65)	1984.65(155.61)	2180.45(116.65)
**2008**	319.59(64.04)	78.33(31.35)	196.11(35.27)		2422.16(176.12)	1229.24(124.11)	1796.01(106.66)		2741.75(187.35)	1307.57(128.00)	1992.12(112.32)
**2009**	316.13(63.41)	360.48(67.50)	328.44(45.63)		2082.18(162.59)	2213.06(167.08)	2143.18(116.45)		2398.31(174.47)	2573.54(180.14)	2471.62(125.04)
**2010**	438.70(74.46)	440.46(74.65)	436.83(52.55)		2034.00(160.19)	944.94(109.32)	1490.52(97.02)		2472.69(176.59)	1385.41(132.33)	1927.36(110.31)
**2011**	124.89(39.73)	77.88(31.42)	109.29(26.30)		1221.21(124.18)	882.61(105.72)	1050.19(81.48)		1346.10(130.36)	960.49(110.28)	1159.48(85.62)
**2012**	187.45(48.55)	299.76(61.75)	237.23(38.73)		1339.39(129.71)	1485.22(137.36)	1419.80(94.70)		1526.84(138.48)	1784.98(150.56)	1657.02(102.29)
**2013**	212.52(51.12)	415.11(71.84)	304.18(43.37)		3041.05(193.11)	1971.07(156.56)	2507.15(124.36)		3253.55(199.72)	2386.18(172.07)	2811.32(131.67)
**2014**	462.75(75.71)	153.13(43.34)	319.39(44.37)		1106.10(117.01)	1721.59(145.22)	1425.47(93.68)		1568.85(139.32)	1874.72(151.52)	1744.86(103.63)
**2015**	126.40(40.43)	222.26(53.44)	177.41(33.82)		1334.55(131.31)	666.78(92.55)	1019.18(81.02)		1460.94(137.38)	889.04(106.85)	1196.59(87.78)
**2016**	250.28(56.46)	301.49(61.85)	285.11(42.57)		2403.91(174.78)	1399.65(133.18)	1888.90(109.48)		2875.36(191.11)	1838.92(152.62)	2349.35(122.07)
**1992-** **2016**	-3.2(-6.9,0.6)	1.3(-2.3,4.8)	-1.4(-5.4,2.6)		4.5(2.6,6.5)*	6.8(2.8,10.7)*	5.5(3.3,7.7)*		2.7(0.9,4.6)*	5.0(2.1,7.9)*	3.7(1.4,6.0)*
**2008-** **2016**	-5.1(-20.2,9.9)	5.8(-15.4,27.1)	0.1(-13.4,13.6)		-2.8(-14.3,8.7)	-1.8(14.6,11.1)	-2.1(-11.8,7.7)		-2.2(-13.1,8.7)	-0.5(-12.6,11.6)	-1.4(-11.3,8.8)

All data was presented as rate (standard error). Abbreviation: ICH=intracerebral hemorrhage, IS=ischemic stroke. *P< 0.05.

### Proportion of first-ever stroke in elderly patients by sex and subtypes

From 1992 to 2016, the proportion of elderly stroke patients in the entire stroke population decreased from 84.2% to 52.4% overall: 81.8% to 50.7% for IS; and 87.5% to 50.0% for ICH. The proportion of elderly patients with first-ever stroke dropped by an average of 1.1% (95% CI: -1.8, -0.4; P<0.05) per year overall: 0.8% (95% CI: -1.5, -0.1; P<0.05) for IS; and 1.8% (95% CI: -2.8, -0.9; P<0.05) for ICH. The corresponding rates were 1.5% (95% CI: -2.2, -0.8) overall: 1.1% (95% CI: -1.8, -0.4) for IS; and 2.7% (95% CI: -1.0, -1.5) for ICH. The decline in the proportion of elderly stroke patients was not statistically significant in women (all P>0.05; Table 3).

**Table 3:** The proportion of elderly stroke in the first-ever stroke by sex and subtypes.

**Year**	**ICH**				**IS**					**Total**	
	**Men**	**Women**	**Total**		**Men**	**Women**	**Total**		**Men**	**Women**	**Total**
**1992**	5(100.0)	2(66.7)	7(87.5)		5(83.3)	4(80.0)	9(81.8)		10(90.9)	6(75.0)	16(84.2)
**1993**	6(100,0)	2(66.7)	8(88.9)		7(70.0)	2(50.0)	9(64.3)		13(81.3)	4(57.1)	17(73.9)
**1994**	2(66.7)	0	2(66.7)		7(87.5)	7(87.5)	14(87.5)		9(81.8)	7(87.5)	16(84.2)
**1995**	3(75.0)	1(33.3)	4(57.1)		5(35.7)	5(45.5)	10(40.0)		8(44.4)	6(42.9)	14(43.8)
**1996**	5(55.6)	1(20.0)	6(42.9)		7(53.8)	5(55.6)	12(54.5)		12(54.5)	6(42.9)	18(50.0)
**1997**	3(75.0)	0	3(60.0)		6(50.0)	0	6(35.3)		9(56.3)	0	9(40.9)
**1998**	4(100.0)	1(33.3)	5(71.4)		8(66.7)	1(25.0)	9(56.3)		12(75.0)	2(28.6)	14(60.9)
**1999**	6(100.0)	1(50.0)	7(87.5)		7(58.3)	1(25.0)	8(50.0)		13(72.2)	2(33.3)	15(62.5)
**2000**	1(50.0)	2(66.7)	3(60.0)		13(72.2)	8(61.5)	21(67.7)		14(70.0)	10(62.5)	24(66.7)
**2001**	2(50.0)	2(100.0)	4(66.7)		10(66.7)	5(71.4)	15(68.2)		12(63.2)	7(77.8)	19(76.9)
**2002**	1(25.0)	4(57.1)	5(45.5)		7(53.8)	5(45.5)	12(50.0)		8(47.1)	9(50.0)	17(48.6)
**2003**	1(12.5)	4(57.1)	5(33.3)		15(55.6)	9(90.0)	24(64.9)		16(45.7)	13(76.5)	29(55.8)
**2004**	5(71.4)	3(60.0)	8(66.7)		10(58.8)	8(57.1)	18(58.1)		15(62.5)	11(57.9)	26(60.5)
**2005**	1(25.0)	1(50.0)	2(33.3)		14(82.4)	7(58.3)	21(72.4)		15(71.4)	8(57.1)	23(65.7)
**2006**	1(20.0)	0	1(14.3)		6(40.0)	3(33.3)	9(37.5)		7(35.0)	3(27.3)	10(32.3)
**2007**	7(70.0)	4(50.0)	11(61.1)		15(60.0)	15(53.6)	30(56.6)		22(62.9)	19(52.8)	41(57.7)
**2008**	3(33.3)	1(25.0)	4(30.8)		22(57.9)	11(57.9)	33(57.9)		25(53.2)	12(52.2)	37(52.9)
**2009**	3(25.0)	3(42.9)	6(31.6)		19(59.4)	21(67.7)	40(63.5)		22(50.0)	24(63.2)	46(56.1)
**2010**	4(44.4)	4(44.4)	8(44.4)		18(48.6)	8(53.3)	26(50.0)		22(47.8)	12(50.0)	34(48.6)
**2011**	1(9.1)	1(33.3)	2(14.3)		11(37.9)	8(33.3)	19(35.8)		12(30.0)	9(33.3)	21(31.3)
**2012**	2(22.2)	3(30.0)	5(26.3)		12(44.4)	14(58.3)	26(51.0)		14(38.9)	17(50.0)	31(44.3)
**2013**	2(25.0)	3(60.0)	5(38.5)		27(45.8)	16(53.3)	43(48.3)		29(43.3)	19(54.3)	48(47.1)
**2014**	4(57.1)	2(40.0)	6(50.0)		10(41.7)	17(60.7)	27(51.9)		14(45.2)	19(57.6)	33(51.6)
**2015**	1(33.3)	2(50.0)	3(42.9)		11(37.9)	6(37.5)	17(37.8)		12(37.5)	8(40.0)	20(38.5)
**2016**	2(33.3)	3(75.0)	5(50.0)		22(56.4)	13(43.3)	35(50.7)		26(55.3)	17(48.6)	43(52.4)
**Total**	-2.7(-4.0,-1.5)*	-0.8(-3.0,1.5)	-1.8(-2.8,-0.9)*		-1.1(-1.8,-0.4)*	-0.5(-1.8,0.8)	-0.8(-1.5,-0.1)*		-1.5(-2.2,-0.8)*	-0.2(-1.3,0.8)	-1.1(-1.8,-0.4)*	

All data was presented as case (%). *P< 0.05.

### Annual trends in the incidence of first-ever stroke in elderly patients compared to young patients from 1992 to 2016 by subtypes

Between 1992 and 2016, the incidence of first-ever stroke in patients aged <65 years increased by 9.5% (men, 10.3%; women, 8.9%) annually and were greater than that in patients aged ≥65 years by 3.7% (men, 2.7%; women, 5.0%). There was a similar trend for IS (total, 10.0% vs. 5.5%; men, 9.9% vs. 4.5%; women, 9.5% vs. 6.8%; all P<0.05). In contrast to findings in elderly patients, the incidence of ICH increased by 9.1% per year for those aged <65 years. Moreover, the increase was significantly higher in women than that in men among patients aged ≥65 years, with a rate of 6.8% vs. 4.5% for IS and 5.0% vs. 2.7% overall (all P<0.05). However, the opposite trend was observed among the younger patients, with a rate of 9.9% vs. 9.5% for IS and 10.3% vs. 8.9% overall (all P<0.05).

**Figure 1:**
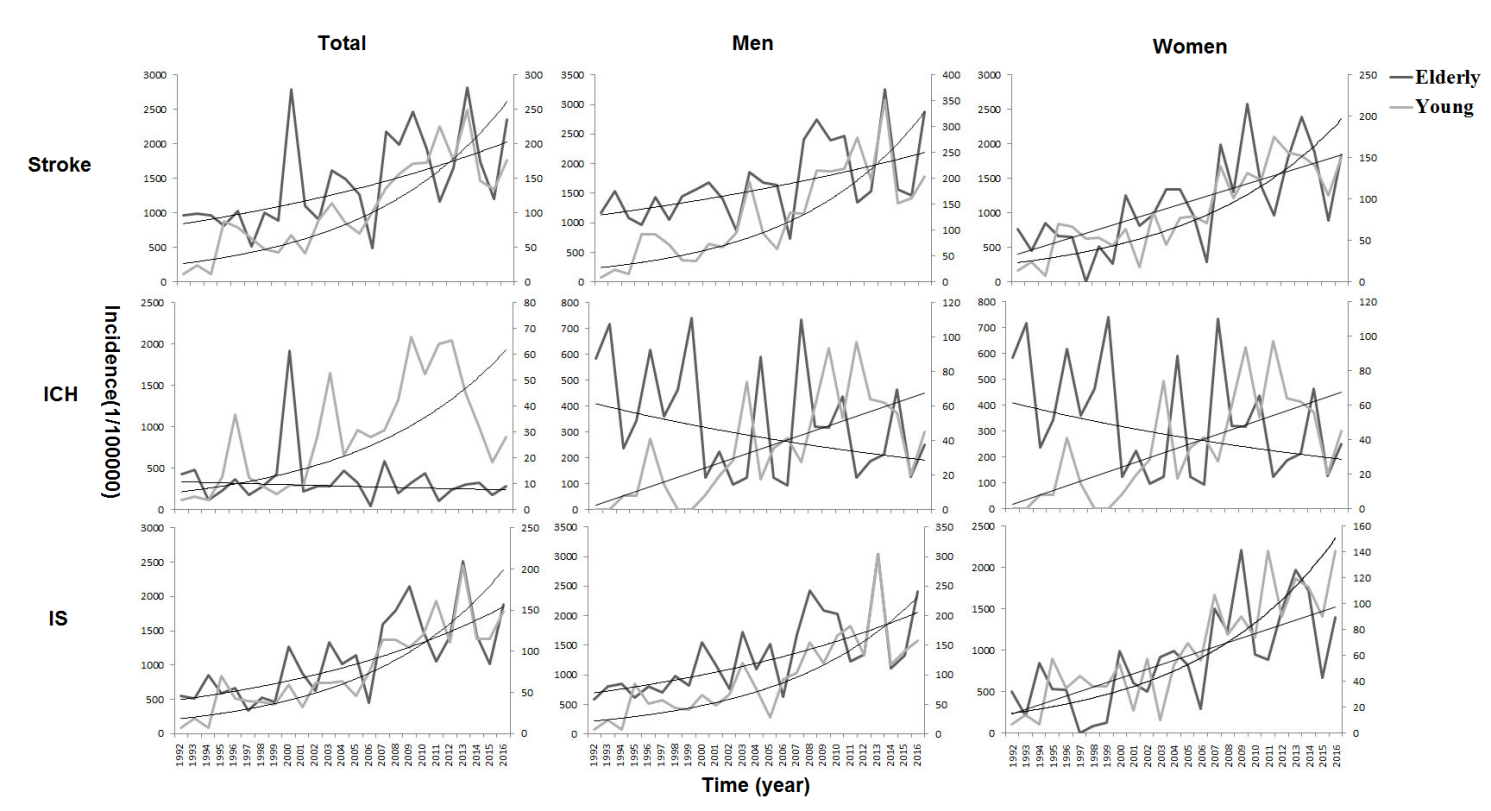
**Annual trends in the incidence of first-ever stroke from 1992 to 2016 by age and subtypes.** tThe increase in stroke incidence was greater in patients aged <65 compared to those aged ≥65 years. In contrast to findings in the elderly population, the incidence of intracerebral hemorrhage (ICH) increased by 9.1% per year for those aged <65 years. Moreover, the increment was significantly higher in women than in men among the patients aged ≥65 years. However, an opposite trend was observed among the younger patients. Over time, the incidence of stroke in the patients aged <65 years continued to increase, while rates of change in the patients aged ≥65 years tended to slow down by degree, both for the total stroke and ischemic stroke (IS).

Over time, the incidence of stroke in the patients aged <65 years continued to increase, while rates of change in the patients aged ≥65 years tended to slow down in degree, both for total stroke and IS (Table 4; Figure 1).

**Table 4:** Trends in incidence of first-ever stroke during 1992 to 2016 by age and subtypes (95% CI).

**Age**	**ICH**				**IS**				**Total**		
	**Men**	**Women**	**Total**		**Men**	**Women**	**Total**		**Men**	**Women**	**Total**
**<65yrs**	7.7 (3.5,11.8)*	1.8 (-1.8,5.4)	9.1 (5.6,12.7)*		9.9 (6.7,13.0)*	9.5 (6.4,12.7)*	10.0 (7.4,12.6)*		10.3 (7.3,13.4)*	8.9 (6.0,11.8)*	9.5 (6.9-12.2)*
**≥65 yrs**	-3.2 (-6.9,0.6)	1.3 (-2.3,4.8)	-1.4 (-5.4,2.6)		4.5 (2.6,6.5)*	6.8 (2.8,10.7)*	5.5 (3.3,7.7)*		2.7 (0.9,4.6)*	5.0 (2.1,7.9)*	3.7 (1.4,6.0)*

*P< 0.05.

## DISCUSSION

This is the first study to report the long-term trends in stroke incidence and stroke subtypes in a Chinese low-income population aged ≥65 years from 1992 to 2016. In this population-based prospective study, we observed an upward trend in the incidence of first-ever stroke among a rural elderly population in China; however, the proportion of older stroke patients in the overall stroke population showed a decreasing trend. Importantly, the annual trend of change in stroke incidence in the population aged ≥65 years tended to be slight, both for total stroke and IS. Between 1992 and 2016, in contrast with rates observed in the younger population, the increase in stroke incidence was significantly higher in women than that in men, among the population aged ≥65 years.

Previous studies have shown that the incidence of stroke in developed countries has declined over the past few decades [9–11]; however, in developing countries, especially in China, the incidence has increased [12–14]. From 1999 to 2008, the incidence of stroke dropped by 29% in the United Kingdom [15]. In the United States, a cohort study of four communities showed that stroke incidence rates among Whites and Blacks, as well as in men and women, dropped significantly between 1987 and 2011 [16]. In South Korea, from 2006 to 2010, the age-standardized incidence of stroke fell from 173/100,000 to 135/100,000 population [17]. In contrast, the incidence of stroke in China has risen over the past few decades. The annual incidence of first-ever stroke among adults aged 40–74 years increased steadily from 189 cases per 100,000 in 2002 to 379 cases per 100,000 population in 2013, resulting in an average annual increase of 8.3% [18]. From 1992 to 2015, the annual incidence of stroke in this study population increased by 12% [19].

Simultaneously, a study in the United States demonstrated that the incidence of stroke among those aged ≥ 65 years showed a decreasing trend by age [16]. It has been reported that the incidence of IS among 35–64-year-old men and women in the Netherlands increased by 20% and 33%, respectively, between 1997 and 2005, and that there was no change in those aged ≥65 years [20]. A Swedish study reported that from 1987 to 2010, the incidence of IS significantly decreased between the age of 65 and 84 years [21]. In contrast to the findings from Western studies, in this study, the age-standardized incidence of stroke increased by 3.7% per year from 1992 to 2016 in the population aged ≥65 years, and the incidence of IS increased at an annual rate of 5.5%. However, the incidence of ICH has not changed over the past 25 years. The reasons for this may be that there were too few cases of hemorrhagic stroke in this population. Importantly, the results of this study showed that over time, the proportion of elderly stroke patients in the overall stroke population was declining. The same trend existed for both IS and ICH. This result showed that the annual trends of change in stroke incidence in the population aged ≥65 years tended to be only slight.

The reason for this phenomenon may be changes in the risk factors for stroke. From 1991 to 2011, in this study population, the prevalence of hypertension in those aged 45–64 years increased by 33%, while the prevalence of hypertension in patients aged ≥65 years did not change. A similar trend has been observed in the consumption of alcohol. The current prevalence of smoking has declined over the past two decades, but an increase of 58% was observed among people <45 years of age [22]. In addition, our previous study showed that the rate of hypertension awareness increased significantly in patients aged 55–64 years, but control and treatment rates increased to a far greater degree in patients aged 65–74 years from 1991 to 2011 [23].

A Swedish study showed that if the incidence of stroke remains constant, the absolute incidence of stroke will increase by 70% and 50% in men and women, respectively, by 2050 [24]. In this study, from 1992 to 2016, the age-standardized incidence of stroke among men aged ≥65 years was higher than that in women, almost every year; however, the increase in stroke incidence among older women was higher than that in men. This phenomenon differs from the findings observed in the younger population (<65 years). A previous study in this population showed that the incidence of stroke among men aged 45–64 years increased at a higher rate than that among women [19].

In elderly women, the higher annual percentage of change may in part be associated with the 12.8-fold increase in the prevalence of diabetes mellitus among these elderly women, from 1991 to 2011 [25]. Additionally, this native neuroprotection is lost within ten years of menopause [26,27]; this may partly explain the increased stroke incidence among elderly women.

Moreover, in this study population, the average level of education of older women was significantly lower than that of men. Lower educational level is also associated with increased stroke risk in elderly women; it is partially mediated by known risk factors, particularly lifestyle and biological factors [28].

There are several limitations to this study. First, the study population was drawn from a town in northern China that was not representative of the entire population. However, because this study focused on trends in the incidence of first-ever stroke among low-income Chinese elderly population, the large population study design and long study period might have reduced the impact of this on the results of this study. In line with the stroke guidelines, the study population met the criteria of ≥100,000 person-years observation period [29]. Second, the proportion of CT/MRI diagnoses (67.3%) among stroke patients aged ≥65 years was less than the recommended 80%, between 1992 and 2016. However, we assessed symptomatic stroke in this study, and all stroke events were verified by a senior neurologist from Tianjin Medical University General Hospital. Finally, when the incidence rates observed in this study were compared with those in other studies, the standardized assessment of the incidence in different populations might have affected the accuracy of the comparison. However, in this study, we discussed the trends in stroke incidence rather than the absolute incidence of stroke and this may reduce the impact of the differences in the reference populations.

## CONCLUSIONS

This is the first report to demonstrate the long-term trends in the incidence of stroke among those aged ≥65 years in a low-income, low-educational population in Tianjin, China. The results of this study showed that, over time, the annual trend of change in stroke incidence in this elderly population tended to be only slight. However, in contrast to trends observed in a younger population, the incidence of stroke in older women was significantly higher than that in older men. These findings indicate that it is crucial to control the risk factors among elderly people, especially among older women, to reduce the burden of stroke in China.

## MATERIALS AND METHODS

### Study population

This study was part of the Tianjin Brain Study, and the study population and design have been described previously [19,22,23,25,30–33]. In 1985, we selected the population of Yangjinzhuang to monitor the epidemiological trends of stroke in Tianjin, China. Stroke events and stroke-related deaths have been recorded since 1985. This population included 15,438 people in 1985, 95% of whom were low-income farmers, distributed among 18 administrative villages. The main source of income was cereal crop production, with a per capita income <100 US dollars in 1990 and <2000 US dollars in 2015 [34]. In 1991, the illiteracy rate among residents aged 35 to 74 years was 30% for men and 40% for women. Demographic characteristics remained stable during the study period [23].

In this study, we analyzed the incidence of the first-ever stroke beginning from 1992, when new diagnostic imaging techniques became available for this population.

The investigative protocol was approved by the ethics committee of Tianjin Medical University General Hospital, and a written informed consent was obtained from each family member.

### Stroke surveillance and quality control

During the periods of surveillance, all stroke events and all-cause deaths were recorded and followed up. Stroke events were reported according to predefined procedures, which included dead patients. Local licensed village physicians reported initial stroke events to the community hospital within 24 hours of onset. Then, community hospital physicians visited the surviving patients’ homes to obtain information on clinical features, and stroke events were confirmed within 72 hours. They reported confirmed stroke events (diagnosis by imaging) monthly to Tianjin Medical University General Hospital, and suspected events (no imaging performed) were reported in a timely manner. The diagnostic review group, including five senior neurologists from Tianjin Medical University General Hospital, confirmed the suspected cases by door-to-door interview, as soon as possible.

Furthermore, changes in all demographic information were registered, including births, deaths, immigrations (due to marriages), and emigrations (due to entry to high school, university, or work in the city). However, peasant workers were included in this study because all residents working in cities are seasonal workers who return to townships at least four times a year.

### Definition of stroke events

First-ever stroke was defined as the first occurrence (no history of stroke in prior medical records) of rapidly developing signs of focal neurologic disturbance of presumed vascular etiology lasting >24 hours [35]. All stroke events were symptomatic strokes and were diagnosed using pre-defined clinical features and imaging evidence. Stroke events included ICH, IS, and unknown. IS was defined as thrombotic cerebral infarction, cardioembolic stroke, or lacunar infarct. In this study, those patients with subarachnoid hemorrhage, transient ischemic attacks, suspected stroke deaths without imaging evidence or confirmation by a Tianjin Medical University General Hospital neurologist, and silent stroke detected only by imaging were excluded. All patients who were confirmed to have stroke underwent alternative CT or MRI examination at a county center hospital. ICH and IS were analyzed in this study.

### Statistical analysis

Stroke incidence was analyzed separately for the time periods 1992 to 2006, and 2007 to 2016. Age-standardized incidence rates of first-ever stroke were calculated assuming a Poisson distribution, using the direct method, standardized according to the world standard population [33]. Age-specific stroke incidence during the study periods was estimated for two age groups: <65 years and ≥65 years. Trends in the age-standardized incidence of stroke were expressed as the annual percentage of change using the regression model log (rt)=a+bt, where log denoted the natural logarithm and t the year. The trend b was estimated from ordinary regression, and 100b represented the estimated annual percentage of change of incidence [36]. Statistical significance was defined as P<0.05. SPSS version 19.0 for Windows (SPSS Inc., Chicago, IL) was used for the analyses.
